# Repeatability of hypoxia PET imaging using [^18^F]HX4 in lung and head and neck cancer patients: a prospective multicenter trial

**DOI:** 10.1007/s00259-015-3100-z

**Published:** 2015-07-02

**Authors:** Catharina M. L. Zegers, Wouter van Elmpt, Katrin Szardenings, Hartmuth Kolb, Alan Waxman, Rathan M. Subramaniam, Dae Hyuk Moon, Jacqueline C. Brunetti, Shyam M. Srinivas, Philippe Lambin, David Chien

**Affiliations:** Department of Radiation Oncology (MAASTRO), GROW - School for Oncology and Developmental Biology, Maastricht University Medical Centre, Maastricht, The Netherlands; Threshold Pharmaceuticals, 170 Harbor Way, South San Francisco, CA 94080 USA; Siemens Molecular Imaging Biomarker Research, Siemens Medical Solutions USA, Inc., 6100 Bristol Parkway, Culver City, CA USA; Cedars-Sinai Medical Center, Los Angeles, CA USA; Boston University School of Medicine, Boston, MA USA; Department of Nuclear Medicine, Asan Medical Center, University of Ulsan College of Medicine, Seoul, Republic of Korea; Holy Name Medical Center, Teaneck, NJ USA; Department of Nuclear Medicine, Imaging Institute, Cleveland Clinic, Cleveland, OH 44195 USA; Division of Nuclear Medicine, Russell H Morgan Department of Radiology and Radiologic Sciences, Johns Hopkins Medical Institutions, Baltimore, MD USA

**Keywords:** PET imaging, HX4, Hypoxia, Head and neck cancer, Lung cancer

## Abstract

**Purpose:**

Hypoxia is an important factor influencing tumor progression and treatment efficacy. The aim of this study was to investigate the repeatability of hypoxia PET imaging with [^18^F]HX4 in patients with head and neck and lung cancer.

**Methods:**

Nine patients with lung cancer and ten with head and neck cancer were included in the analysis (NCT01075399). Two sequential pretreatment [^18^F]HX4 PET/CT scans were acquired within 1 week. The maximal and mean standardized uptake values (SUV_max_ and SUV_mean_) were defined and the tumor-to-background ratios (TBR) were calculated. In addition, hypoxic volumes were determined as the volume of the tumor with a TBR >1.2 (HV_1.2_). Bland Altman analysis of the uptake parameters was performed and coefficients of repeatability were calculated. To evaluate the spatial repeatability of the uptake, the PET/CT images were registered and a voxel-wise comparison of the uptake was performed, providing a correlation coefficient.

**Results:**

All parameters of [^18^F]HX4 uptake were significantly correlated between scans: SUV_max_ (*r* = 0.958, *p* < 0.001), SUV_mean_ (*r* = 0.946, *p* < 0.001), TBR_max_ (*r* = 0.962, *p* < 0.001) and HV_1.2_ (*r* = 0.995, *p* < 0.001). The relative coefficients of repeatability were 15 % (SUV_mean_), 17 % (SUV_max_) and 17 % (TBR_max_). Voxel-wise analysis of the spatial uptake pattern within the tumors provided an average correlation of 0.65 ± 0.14.

**Conclusion:**

Repeated hypoxia PET scans with [^18^F]HX4 provide reproducible and spatially stable results in patients with head and neck cancer and patients with lung cancer. [^18^F]HX4 PET imaging can be used to assess the hypoxic status of tumors and has the potential to aid hypoxia-targeted treatments.

**Electronic supplementary material:**

The online version of this article (doi:10.1007/s00259-015-3100-z) contains supplementary material, which is available to authorized users.

## Introduction

[^18^F]HX4 is a new 2-nitroimidazole PET imaging agent for hypoxia, in which structure–activity relationships have been used to optimize pharmacokinetic and clearance properties [1, 2]. Tumor hypoxia is a condition in which insufficiently vascularized tumor cells deprived of oxygen not only become more aggressive and malignant, but also more resistant to treatment by radiation and chemotherapy [3–5]. The presence of hypoxia is therefore generally considered a poor prognostic disease marker in cancer patients [6]. However, it is difficult to measure oxygen levels reproducibly and noninvasively in a highly heterogeneous tumor environment. Reliable diagnostic methods to detect and quantify tumor hypoxia are therefore needed. It has been hypothesized and currently being investigated that inclusion of hypoxic cell sensitizers during treatment, i.e., the delivery of higher radiotherapy doses to hypoxic regions [7] or the use of hypoxia-targeting therapy [8–11], might improve the outcome in patients with hypoxic tumors [12]. [^18^F]HX4 has the potential to serve as a clinically useful diagnostic tool to aid the use of hypoxia-targeting therapies in those patients who will most likely benefit from them [13, 14].

This pilot phase 2 study was primarily designed as a test–retest study to investigate the repeatability of [^18^F]HX4 as a noninvasive PET imaging marker for detection of tumor hypoxic regions. Here we present the results in patients with lung cancer and patients with head and neck (H&N) cancer.

## Materials and methods

### Patients

This multicenter study (NCT01075399) was conducted in accordance with the ethical principles of Good Clinical Practice, according to the International Conference on Harmonization of Technical Requirements for Registration of Pharmaceuticals for Human Use (ICH). Both the FDA and the institutional review boards of the participating institutions approved the study protocol and the informed consent form. All participants reviewed and signed the informed consent form before study entry. [^18^F]HX4 PET/CT images were acquired in 19 patients, 9 with lung cancer and 10 with H&N cancer. The patients underwent two sequential pretreatment [^18^F]HX4 PET/CT scans within 1 week to assess repeatability. Patient characteristics are presented in Table 1.

**Table 1:** Patient characteristics

Patient ID	Age (years)	Weight (kg)	Gender	Lesion location	TNM stage	Pathology	Gross tumor volume (cm^3^)
Lung cancer							
01	67	73	F	RUL lung	T4N2M1	Adenocarcinoma	88.8
02	54	78	M	LUL lung	T4N3M1	Small-cell carcinoma	361.5
03	65	68	M	R precarina	T1N3M0	Large-cell carcinoma	5.2
04	71	57	F	R mediastinum	T3N2M0	Large-cell carcinoma	251.6
05	66	68	F	RLL lung	T2N2M0	Adenocarcinoma	87.6
06	60	65	M	RUL lung	T4N3M0	Squamous cell carcinoma	23.0
07	61	84	M	RUL lung	T2aN2M1	Adenocarcinoma	10.2
08	62	71	F	RUL lung	T2N0M0	Adenocarcinoma	9.2
09	68	84	F	LUL lung	T1bN0M0	Large-cell carcinoma	4.1
Head and neck cancer							
10	46	98	M	R neck lymph node	T1N1M0	NA	20.5
11	60	61	F	Anterior larynx	T3N2cM0	Squamous cell carcinoma	7.0
12	65	79	F	L soft palate	T4N0M0	Squamous cell carcinoma	79.9
13	58	84	M	R base of tongue	T2N2aM0	Squamous cell carcinoma	2.6
14	71	82	M	R neck	T2N2bM0	Squamous cell carcinoma	17.8
15	61	64	M	L aryepiglottic fold	T2N2aM0	Squamous cell carcinoma	31.6
16	53	118	M	R piriform sinus	T1N1M0	Squamous cell carcinoma	6.9
17	40	54	F	R maxillary sinus	T4N2M0	Adenoid cystic carcinoma	248.1
18	63	82	M	R base of tongue	T1N2bM0	Squamous cell carcinoma	5.3
19	64	98	M	R sinonasal space	T4aN0M0	Undifferentiated carcinoma	68.3

*RUL* right upper lobe, *LUL* left upper lobe, *RLL* right lower lobe, *R* right, *L* left, *NA* not available

### Radiochemistry

[^18^F]HX4 (flortanidazole, 3-[^18^F]fluoro-2-(4-((2-nitro-1H-imidazol-1-yl)methyl)-1H-1,2,3-triazol-1-yl)-propan-1-ol) was prepared by Siemens Molecular Imaging (Culver City, CA) or a Siemens PETNET qualified manufacturing site and delivered to each site on the day of injection. The radiosynthesis has been described previously [15]. Briefly, the precursor (Siemens Molecular Imaging Inc., Culver City, California, USA) was reacted with ^18^F-K2.2.2 and K_2_CO_3_ in MeCN at 110 °C for 10 min, followed by a deprotection step using 1.0 mol/l HCl at 100 °C for 5 min. [^18^F]HX4 was purified by RP-HPLC and stabilized with ascorbic acid before sterile filtration. In order to be released, each dose of [^18^F]HX4 had to have a radiochemical purity greater than 95 %.

### Scanners and technical parameters

[^18^F]HX4 PET/CT scans were performed using a high-resolution full-ring PET/CT scanners, including a GE Discovery, GE Discovery LS, Philips Gemini, and a Siemens Biograph PET/CT scanner. Images were reconstructed using scanner-specific parameters in accordance with each facility’s standard procedure, including at least attenuation and scatter correction. Repeat scans were performed on the same PET/CT scanner using the same protocol and patient positioning without respiratory gating.

### [^18^F]HX4 PET/CT imaging

For each [^18^F]HX4 PET/CT scan, the patient received a single intravenous bolus injection of 368 ± 48 MBq (range 199 – 488 MBq) of [^18^F]HX4 followed by a saline flush. A static PET/CT scan was acquired with an acquisition time of 3 min (range 1.7 – 5 min) per bed position after an uptake time of 99 ± 10 min (range 89 – 125 min). The average difference in uptake time between repeat PET scans was 6 ± 7 min (range 0 – 27 min).

### Image evaluation of [^18^F]HX4

[^18^F]HX4 PET/CT scans were analyzed using an Inveon Research Workplace (Edition 4.0.0.3; Siemens, Germany). Gross tumor volumes (GTV) of the primary lesion or largest lymph node were defined in centimeters cubed by manual contouring of the tumor on the CT images by one observer (D.C.). These tumor delineations were applied to the PET images and the maximal and mean standardized uptake values (SUV_max_, SUV_mean_) were measured in grams per milliliter. Under the assumption of water density, the SUV is reported as unitless. For each patient, the reference tissue was defined by contouring a volume of interest (VOI; sphere of radius 25 mm) in a large (thigh) muscle on the CT image. From this muscle VOI the SUV_mean_ (M) was determined. Tumor-to-background ratios (TBR) were calculated by dividing tumor SUV_max_ and tumor SUV_mean_ by muscle SUV_mean_ (M)

$$ \begin{array}{c}\hfill {\mathrm{TBR}}_{\max } = \mathrm{Tumor}\ {\mathrm{SUV}}_{\max }/\ \mathrm{M}\hfill \\ {}\hfill {\mathrm{TBR}}_{\mathrm{mean}} = \mathrm{Tumor}\ {\mathrm{SUV}}_{\mathrm{mean}}/\ \mathrm{M}\hfill \end{array} $$

The hypoxic volume (HV; in centimeters cubed) of each tumor was defined as the [^18^F]HX4 tumor volume with a TBR >1.2 (HV_1.2_) or TBR >1.4 (HV_1.4_):

$$ \begin{array}{c}\hfill {\mathrm{HV}}_{1.2} = \mathrm{V}\mathrm{olume}\ \mathrm{with}\mathrm{in}\ \mathrm{G}\mathrm{T}\mathrm{V}\ \mathrm{with}\ \mathrm{T}\mathrm{B}\mathrm{R} > 1.2\hfill \\ {}\hfill {\mathrm{HV}}_{1.4} = \mathrm{V}\mathrm{olume}\ \mathrm{with}\mathrm{in}\ \mathrm{G}\mathrm{T}\mathrm{V}\ \mathrm{with}\ \mathrm{T}\mathrm{B}\mathrm{R} > 1.4\hfill \end{array} $$

The fraction of HV (FHV, percent) of each tumor was determined by dividing the HV by its respective GTV:

$$ \begin{array}{c}\hfill {\mathrm{FHV}}_{1.2} = {\mathrm{HV}}_{1.2}/\ \mathrm{G}\mathrm{T}\mathrm{V}\hfill \\ {}\hfill {\mathrm{FHV}}_{1.4} = {\mathrm{HV}}_{1.4}/\ \mathrm{G}\mathrm{T}\mathrm{V}\hfill \end{array} $$

To evaluate the repeatability of the heterogeneous uptake pattern, the second [^18^F]HX4 PET/CT scans were rigidly registered and inspected for accurate registration, and a voxel-wise comparison of the SUVs within the GTV was performed.

### Statistics

For all parameters, the mean ± SD are reported. The relationships among GTV-based parameters (SUV_mean_, SUV_max_, TBR, HV, FHV) extracted from repeat [^18^F]HX4 PET images were analyzed by calculating Pearson correlation coefficients. A *p* value <0.05 was assumed to be statistically significant. In addition, a Bland-Altman analysis was performed for all parameters providing the mean difference of each parameter and the absolute and relative coefficients of repeatability (CR 1.96 × SD), defined as the value below which the difference between two measurements will be with 95 % probability. To evaluate the voxel-wise analysis, a linear fit of the data was performed, providing the correlation coefficient and slope. A Bland-Altman plot was created providing the difference in uptake for each matching voxel (ΔSUV) with the lower and upper limits of agreement of the 95 % confidence interval. In addition a histogram of SUVs within the GTV was prepared.

## Results

[^18^F]HX4 PET/CT imaging in nine patients with lung cancer and ten with H&N cancer were included in the analysis. Two sequential baseline [^18^F]HX4 PET/CT scans were performed at an average interval of 1.1 days (range 1 – 2 days) in patients with lung cancer and 2.1 days (range 1 – 6 days) in patients with H&N cancer.

### [^18^F]HX4 uptake in the GTV

[^18^F]HX4 uptake varied considerably among tumors on both the first scan with an average SUV_max_ of 1.86 ± 0.52 (range  1.2 – 2.9) and SUV_mean_ of 1.20 ± 0.28 (range 0.85 – 1.90) and the second scan with an average SUV_max_ of 1.84 ± 0.50 (range 1.15 – 2.82) and SUV_mean_ of 1.20 ± 0.28 (range 0.92 – 1.97; Table 2).

**Table 2:** Repeatability of [^18^F]HX4 uptake and hypoxic tumor volume and fraction using a threshold of 1.2 times background (HV_1.2_)

Patient ID	SUV_mean_		SUV_max_		TBR_max_		HV_1.2_ (cm^3^)		FHV_1.2_ (%)	
	Scan 1	Scan 2	Scan 1	Scan 2	Scan 1	Scan 2	Scan 1	Scan 2	Scan 1	Scan 2
Lung cancer										
01	1.20	1.09	2.15	2.21	1.72	1.87	14.01	11.15	15.78	11.33
02	1.38	1.49	2.87	2.74	2.40	2.10	147.34	148.31	40.76	38.55
03	1.17	1.19	1.47	1.60	1.20	1.27	0.06	0.26	1.23	9.30
04	1.90	1.97	2.93	2.82	2.03	1.94	177.66	203.33	70.62	74.95
05	1.16	1.10	1.68	1.67	1.50	1.66	18.82	18.50	21.48	36.04
06	0.89	0.90	1.46	1.55	1.49	1.62	2.49	1.80	10.85	9.52
07	0.94	1.03	1.57	1.64	1.47	1.51	0.45	1.45	4.41	9.67
08	1.36	1.51	1.98	2.25	1.64	1.75	3.58	4.58	38.85	47.92
09	1.19	1.07	1.63	1.47	1.17	1.16	0.00	0.00	0.00	0.00
Mean ± SD	1.24 ± 0.29	1.26 ± 0.33	1.97 ± 0.57	1.99 ± 0.53	1.63 ± 0.39	1.65 ± 0.31	40.5 ± 69.9	43.3 ± 76.6	22.6 ± 23.4	26.4 ± 24.6
Head and neck cancer										
10	0.91	0.92	1.25	1.29	1.04	1.01	0.00	0.00	0.00	0.00
11	0.89	0.99	1.23	1.31	1.04	1.08	0.00	0.00	0.00	0.00
12	1.85	1.72	2.52	2.39	1.32	1.27	3.90	1.54	4.89	2.02
13	1.09	0.97	1.30	1.15	1.17	1.09	0.00	0.00	0.00	0.00
14	1.15	0.99	1.71	1.37	1.42	1.28	0.90	0.13	5.04	0.67
15	1.17	1.12	1.72	1.80	1.44	1.69	3.65	5.57	11.56	20.57
16	0.98	1.05	1.77	1.96	1.79	2.08	1.01	2.03	14.55	33.83
17	1.23	1.17	2.48	2.33	3.35	3.15	211.24	203.71	85.14	80.03
18	1.15	1.18	1.49	1.39	1.36	1.23	0.60	0.27	11.33	5.11
19	1.26	1.28	2.12	2.05	1.96	1.99	28.73	41.24	42.07	57.56
Mean ± SD	1.17 ± 0.27	1.14 ± 0.23	1.76 ± 0.48	1.70 ± 0.46	1.59 ± 0.69	1.59 ± 0.67	25.0 ± 66.0	25.5 ± 63.9	17.5 ± 26.9	20.0 ± 28.5
Mean ± SD (total)	1.20 ± 0.28	1.20 ± 0.28	1.86 ± 0.52	1.84 ± 0.50	1.61 ± 0.55	1.62 ± 0.52	32.3 ± 66.4	33.9 ± 68.8	19.9 ± 24.7	23.0 ± 26.2

**Fig. 1:**
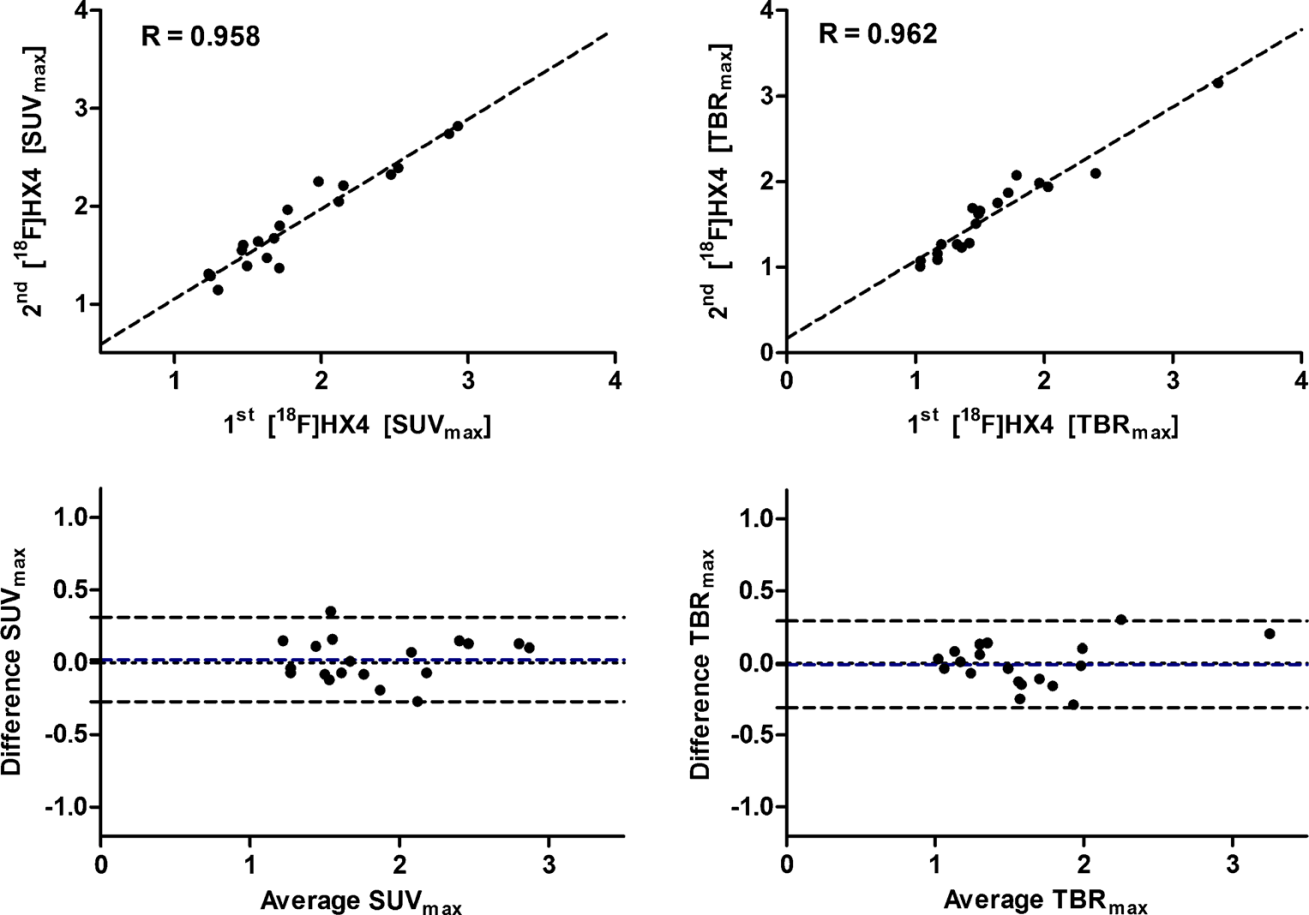
Correlation and Bland-Altman plots (including 95 % confidence intervals) of the image parameters SUV_max_ and TBR_max_

The uptake parameters from the first and second scans were highly correlated: *r* = 0.958 for SUV_max_ (*p* < 0.001, Fig. 1), and *r* = 0.946 for SUV_mean_ (*p* < 0.001, Supplementary figure). High correlations between scans were also seen within each subgroup of cancer patients: *r* = 0.972 for SUV_max_ (*p* < 0.001) and *r* = 0.960 for SUV_mean_ (*p* < 0.001) in those with lung cancer, and *r* = 0.945 for SUV_max_ (*p* < 0.001) and *r* = 0.952 for SUV_mean_ (*p* < 0.001) in those with H&N cancer. In the Bland-Altman analysis, SUV_max_ showed a mean difference of 0.02 with an absolute CR of 0.29 and a repeatability percentage of 17 % (Fig. 1), and SUV_mean_ showed a mean difference of 0.01 with an absolute CR of 0.18 and a repeatability percentage of 15 %.

High correlations were also seen for TBR_max_ (*r* = 0.962, *p* < 0.001; Fig. 1) and TBR_mean_ (*r* = 0.965, *p* < 0.001). High correlations were also seen within each subgroup of cancer patients: *r* = 0.939 for TBR_max_ (*p* < 0.001) and *r* = 0.972 for TBR_mean_ (*p* < 0.001) in those with lung cancer, and similarly *r* = 0.972 for TBR_max_ (*p* < 0.001) and *r* = 0.964 for TBR_mean_ (*p* < 0.001) in those with H&N cancer. In the Bland-Altman analysis, TBR_max_ showed a mean difference of −0.01 with an absolute CR of 0.30 and a repeatability percentage of 17 % (Fig. 1), and TBR_mean_ showed a mean difference of −0.01 with an absolute CR of 0.11 and a repeatability percentage of 10 %.

### HV and FHV analysis

The average tumor volume was 70 cm^3^ (range 2.6 – 361 cm^3^). The average HV_1.2_ in the first scan was 32 cm^3^ (range 0 – 211 cm^3^) and in the second scan was 34 cm^3^ (range 0 – 204 cm^3^; Table 2). For HV_1.2_, there was a high correlation between the first and second scans (*r* = 0.995, *p* < 0.001; Supplementary figure) which was retained in each subgroup of cancer patients: *r* = 0.997 (*p* < 0.001) in those with lung cancer and *r* = 0.998 (*p* < 0.001) in those with H&N cancer. In the Bland-Altman analysis, HV_1.2_ showed a mean difference of –1.55 cm^3^ with an absolute CR of 13.5 cm^3^ (Supplementary figure).

Applying the higher threshold of 1.4 times the background, in the first scan the average HV_1.4_ was 19 cm^3^ (range 0 – 175 cm^3^) and in the second scan was 19 cm^3^ (range 0 – 162 cm^3^; Supplementary table). For HV_1.4_, there was also a consistently high correlation between the first and second scans (*r* = 0.982, *p* < 0.001) which was retained in each subgroup of cancer patients: *r* = 0.959 (*p* < 0.001) in those with lung cancer and *r* = 0.999 (*p* < 0.001) in those with H&N cancer. In the Bland-Altman analysis, HV_1,4_ showed a mean difference of 0.08 cm^3^ with a confidence interval of –17.2 to 17.4 cm^3^.

There was a wide range of FHV_1.2_ due to varying levels of hypoxia among the tumors. In the first scan the average FHV_1.2_ was 20 ± 25 % (range 0 – 85 %) and in the second scan the average FHV_1.2_ was 23 ± 26 % (range 0 – 80 %; Table 2). This was also seen when the higher threshold of 1.4 times the background was applied: in the first scan the average FHV_1.4_ was 9 ± 18 % (range 0 – 71 %) and in the second scan the average FHV_1.4_ was 10 ± 17 % (range 0 – 63 %; Supplementary table).

For FHV_1.2_, there was a high correlation between the first and second scans (*r* = 0.957, *p* < 0.001) which was retained in each subgroup of cancer patients: *r* = 0.966 (*p* < 0.001) in those with lung cancer and *r* = 0.950 (*p* < 0.001) in those with H&N cancer. For FHV_1.4_, there was also a high correlation between the first and second scans (*r* = 0.975, *p* < 0.001) which was retained in each subgroup of cancer patients: *r* = 0.963 (*p* < 0.001) in those with lung cancer and *r* = 0.985 (*p* < 0.001) in those with H&N cancer. In the Bland Altman analysis, FHV_1.2_ showed a mean difference of -3.1 % with an absolute CR of 14.9 %, and FHV_1.4._ showed a mean difference of -0.9 % and an absolute CR of 7.8 %.

Using 1.2 times the background as the threshold to determine FHV, 79 % of the tumors (15/19) were found to have some level of hypoxia but when the higher threshold of 1.4 times the background was applied to determine FHV, only 47 % of the tumors (9/19) were characterized as having hypoxia.

### Repeatability of the spatial uptake pattern

**Fig. 2:**
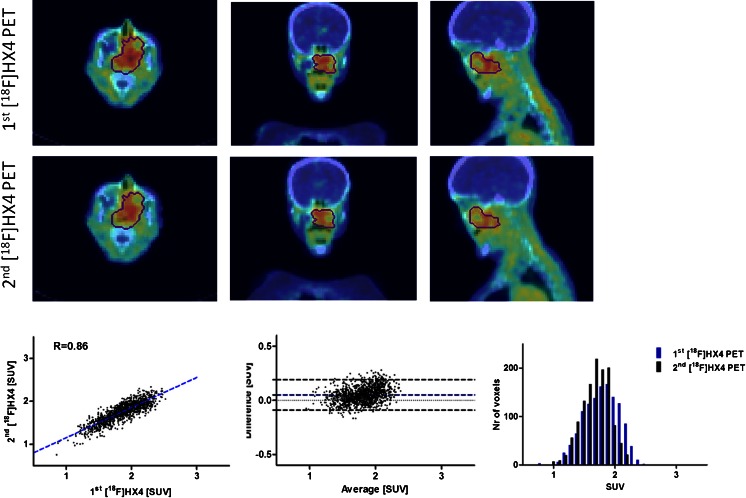
Example of voxel-wise analysis in a patient with head and neck cancer (patient 12). The axial, coronal and sagittal planes of the first and the rigidly registered second [^18^F]HX4 PET/CT scan are shown. The gross tumor volume is delineated. The *bottom row* shows the correlation plot, the Bland-Altman and the histogram plot of the voxels within the gross tumor volume

**Fig. 3:**
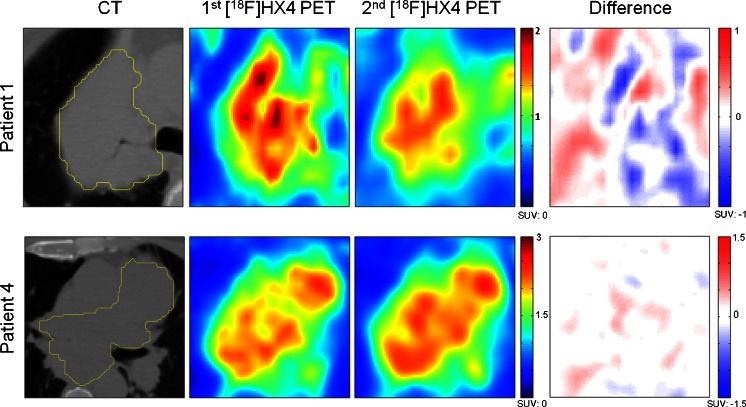
Examples of voxel-wise analysis in patients with lung cancer (patients 1 and 4). The axial plane of the CT scans with the gross tumor volumes delineated in *yellow*, the first [^18^F]HX4 PET scan, the rigidly registered second [^18^F]HX4 PET scan and the difference map from the two scans are shown

An example of voxel-wise image analysis in a patient with head and neck cancer (patient 12) is shown in Fig. 2. Comparison of the heterogeneous uptake within the GTV between the first and second [^18^F]HX4 PET scans showed a moderate to strong correlation in the majority of patients, with an average correlation coefficient of 0.65 ± 0.14. There were two exceptions (patients 14 and 16) in whom a poor correlation was observed (*R* = 0.38 and 0.39). The average slope and intercept of the linear fit of the data were 0.56 ± 0.17 and 0.47 ± 0.19, respectively. The Bland-Altman analysis showed an average ΔSUV of 0.02 ± 0.06, with a lower and upper limit of agreement of 0.15 ± 0.09 and 0.19 ± 0.08. Examples of voxel-wise image analysis in patients with lung cancer (patients 1 and 4) are shown in Fig. 3. In addition, the results for each patient are shown in Table 3.

**Table 3:** Results of the voxel-wise analysis

Patient ID	Correlation plot			Bland-Altman analysis		
	Pearson’s correlation coefficient (*R*)	Slope	Intercept	Mean ΔSUV	95 % confidence interval	
					Lower limit of agreement	Upper limit of agreement
Lung cancer						
01	0.51	0.39	0.64	0.05	−0.21	0.30
02	0.61	0.46	0.85	−0.06	−0.34	0.23
03	0.69	0.72	0.35	−0.01	−0.15	0.13
04	0.85	0.82	0.4	−0.03	−0.20	0.14
05	0.58	0.78	0.02	0.12	−0.10	0.35
06	0.83	0.63	0.27	0.03	−0.10	0.15
07	0.55	0.45	0.62	−0.05	−0.24	0.13
08	0.82	0.86	0.28	−0.04	−0.21	0.13
09	0.62	0.44	0.52	0.07	−0.07	0.21
Mean ± SD	0.67 ± 0.13	0.63 ± 0.18	0.42 ± 0.24	0.02 ± 0.07	−0.19 ± 0.08	0.22 ± 0.12
Head and neck cancer						
10	0.63	0.59	0.35	0.01	−0.12	0.14
11	0.84	0.71	0.31	−0.02	−0.12	0.07
12	0.86	0.70	0.45	0.05	−0.09	0.19
13	0.69	0.30	0.45	0.15	0.06	0.25
14	0.38	0.28	0.61	0.11	−0.07	0.28
15	0.56	0.49	0.52	0.04	−0.12	0.20
16	0.39	0.37	0.68	−0.03	−0.25	0.19
17	0.63	0.61	0.42	0.03	−0.23	0.30
18	0.70	0.49	0.61	−0.01	−0.11	0.09
19	0.63	0.49	0.64	0.00	−0.21	0.21
Mean ± SD	0.63 ± 0.16	0.50 ± 0.15	0.50 ± 0.13	0.03 ± 0.06	−0.13 ± 0.09	0.19 ± 0.07
Mean ± SD (total)	0.65 ± 0.14	0.56 ± 0.17	0.46 ± 0.19	0.03 ± 0.06	−0.16 ± 0.09	0.21 ± 0.10

## Discussion

The aim of this study was to investigate the repeatability of [^18^F]HX4 as a noninvasive PET imaging marker for the detection of tumor hypoxia in patients with lung cancer and patients with H&N cancer. Tumor hypoxia is known to be a dynamic process characterized by the presence of acute and chronic hypoxia. Acute hypoxia is usually the result of a blockage or disruption in the perfusion of the tumor, while chronic hypoxia is mainly caused by limitations of oxygen diffusion due to an inefficient blood vessel network which results in larger distances between the blood vessels and tumor tissue. Static PET imaging will show only the hypoxic status at one specific time-point and contain information about both acute and chronic hypoxia. To be able to select patients for treatment with antihypoxia therapy and/or for a hypoxia-based radiotherapy dose redistribution, it is important to gain an insight into the day-to-day variability in tumor hypoxia and its spatial location. Therefore we compared [^18^F]HX4 uptake, tumor-to-muscle levels and hypoxic fractions between two consecutive [^18^F]HX4 PET scans. To obtain information about the spatial distribution of tumor hypoxia, a voxel-wise comparison of the [^18^F]HX4 uptake was performed.

While there was, as anticipated, a large interpatient variability in [^18^F]HX4 uptake, no major differences were observed between patients with H&N or patients with lung cancer. The average SUV of [^18^F]HX4 was identical for lung cancer (1.2 ± 0.3) and H&N cancer lesions (1.2 ± 0.3). There is no standardized method to define tumor hypoxia on PET images. The threshold value for defining tumor hypoxia is dependent on the tracer, tracer pharmacokinetics, and other imaging parameters [16]. In a previous study [16], we showed that PET imaging using a threshold of 1.2 times background at 2 h after injection provides a similar FHV and hypoxic lesion detection rate to imaging using a threshold of 1.4 times background at 4 h after injection. In the current analysis, we included both thresholds to quantify the HV. First we defined the threshold as an uptake above 1.2 times the background level. In this case 89 % (8/9) of the patients with lung cancer and 70 % (7/10) of those with H&N cancer had a hypoxic tumor volume. These percentages are in agreement with previously published results showing, for example, hypoxia in 72 % of patients with non-small-cell lung cancer [16] and in 84 % of those with H&N cancer [17]. Increasing the threshold to 1.4 times background level resulted in decreases in the proportions of hypoxic lesions detected to 67 % of lung cancer lesions (6/9) and 30 % of H&N cancer lesions (3/10).

At the tumor level we observed a high correlation for the frequently used parameters to quantify tumor hypoxia (SUV_max_, SUV_mean_, TBR, HV and FHV). This is in agreement with the results of a study by Okamoto et al. [18] who evaluated the reproducibility of the hypoxia PET tracer [^18^F]FMISO in patients with H&N cancer. They found a high correlation for SUV_max,_ TBR and HV. However, these results do not agree with the previous results of Nehmeh et al. [19] who found a considerable variability in intratumoral uptake between repeat [^18^F]FMISO PET scans. The reproducibility of the hypoxia PET tracer [^18^F]FAZA was evaluated by Busk et al. [20] in a mouse model and showed good reproducibility. In comparison to [^18^F]FDG PET/CT imaging, our observed repeatability percentages (SUV_max_ 17 % and SUV_mean_ 15 %) are smaller than the relative differences required to exceed test–retest variability, which should be larger than 25 % for SUV_max_ and 20 % for SUV_mean_ [21]. Since [^18^F]HX4 has a lower uptake than [^18^F]FDG, results from comparisons of the two tracers should be interpreted with caution. However, comparing our relative coefficients of repeatability with the results of the low uptake [^18^F]FDG measurements (Fig. 1c of de Langen et al. [21]), the observed [^18^F]HX4 repeatability percentage of the SUV_max_ (17 %) is much lower than expected based on [^18^F]FDG (approximately 35 %). This high repeatability of [^18^F]HX4 PET imaging parameters at the tumor level provides confidence that hypoxia PET imaging using [^18^F]HX4 can be used to reliably detect and quantify tumor hypoxia. This is essential for the use of hypoxia PET imaging as a predictor of treatment response or for monitoring changes in hypoxia during treatment. The detection of hypoxia using [^18^F]HX4 PET/CT at the tumor level could therefore be used to identify patients who might benefit from hypoxia-targeted treatment [22].

To evaluate the stability of the heterogeneous uptake pattern of [^18^F]HX4, a voxel-wise comparison was performed. This analysis showed reproducible results (*R* > 0.5) in the majority (17 out of 19) patients with lung cancer or H&N cancer. The observed repeatability is in agreement with previous results of Peeters et al. [23] showing high repeatability of [^18^F]HX4 uptake in a rat rhabdomyosarcoma model. Repeatability studies using the alternative hypoxia tracer [^18^F]FMISO have shown contradictory results: Okamoto et al. [18] and Bittner et al. [24] found good repeatability, while Nehmeh et al. [19] observed variability in spatial uptake. For the hypoxia tracer [^18^F]FAZA, repeated PET/CT imaging was performed during the course of radiotherapy. While Mortensen et al. [25] found a stable location of the HV during treatment, Servagi-Vernat et al. [26] found a spatial move in the HV. The spatial reproducibility of tumor hypoxia, as measured by a hypoxia PET tracer is essential for hypoxia PET-based radiotherapy planning. Three-dimensional information on the hypoxic areas within the tumor can be used to tailor radiotherapy treatment to give a higher radiation dose to hypoxic subvolumes [27]. In this study, [^18^F]HX4 PET/CT imaging was able to identify stable hypoxic areas in the majority of patients. Therefore, this imaging technique could potentially enable the reliable treatment of hypoxic areas with an increased radiotherapy dose. Several studies have already shown that it is feasible to perform radiotherapy dose planning based on hypoxia PET images [12, 28, 29].

There were some limitations to this study. First, patients with very heterogeneous disease were included. These tumors have a different histology and might therefore express a different phenotype regarding acute versus chronic tumor hypoxia, which could possibly affect the reproducibility of tracer uptake. Nevertheless, even in this heterogeneous population, a high repeatability in [^18^F]HX4 PET/CT uptake was observed. Second, the study design was multicentric; therefore different PET/CT scanners were used with different physical characteristics and different acquisition protocols. Differences in resolution among the scanners might have led to differences in the tumor hypoxia detection rates. In general, we expect with all scanners a partial volume effect, and particularly in small lesions, in lesions with low uptake and with a small HV this would cause larger differences in absolute uptake measurements. Also, breathing motion in the patients with lung cancer could have caused blurring of the PET signal. The differences in acquisition protocol, i.e., acquisition time per bed position and uptake period, will lead to differences in the observed signal-to-noise ratios, and TBR and SUV measurements [16, 30]. Nevertheless, since we used each patient as his or her own control, the partial volume effect and the effect of different scanners should have had only a minor influence on the repeatability results. Third, the [^18^F]HX4 PET scans were on average acquired at 99 min after injection, with a maximal difference in the time from injection acquisition of 27 min. Studies reported after this study was completed have shown that the contrast between tumor and background increases up to 4 h after injection. Therefore, the image contrast might have been suboptimal and the differences in uptake parameters observed might have been due to differences in the time from injection to acquisition [30].

In conclusion, repeated PET imaging with the hypoxia tracer [^18^F]HX4 provides reliable and reproducible results regarding the (spatial) uptake in patients with head and neck and lung cancer. [^18^F]HX4 has the potential to quantify hypoxia in tumors and aid hypoxia-targeted treatments.

## Electronic supplementary material

Supplementary Figure 1 Correlation and Bland-Altman plots of image parameters SUV_mean_ and hypoxic tumor volume (HV_1.2_). (JPEG 740 kb)

Supplementary Table 1 (DOC 72 kb)
