# Supplementary figures and images for: Repeatability of hypoxia PET imaging using [18F]HX4 in lung and head and neck cancer patients: a prospective multicenter trial
Source: Eur J Nucl Med Mol Imaging. 2015 Jul 2;42(12):1840–9. doi: 10.1007/s00259-015-3100-z (PMC4589564; doi:10.1007/s00259-015-3100-z)

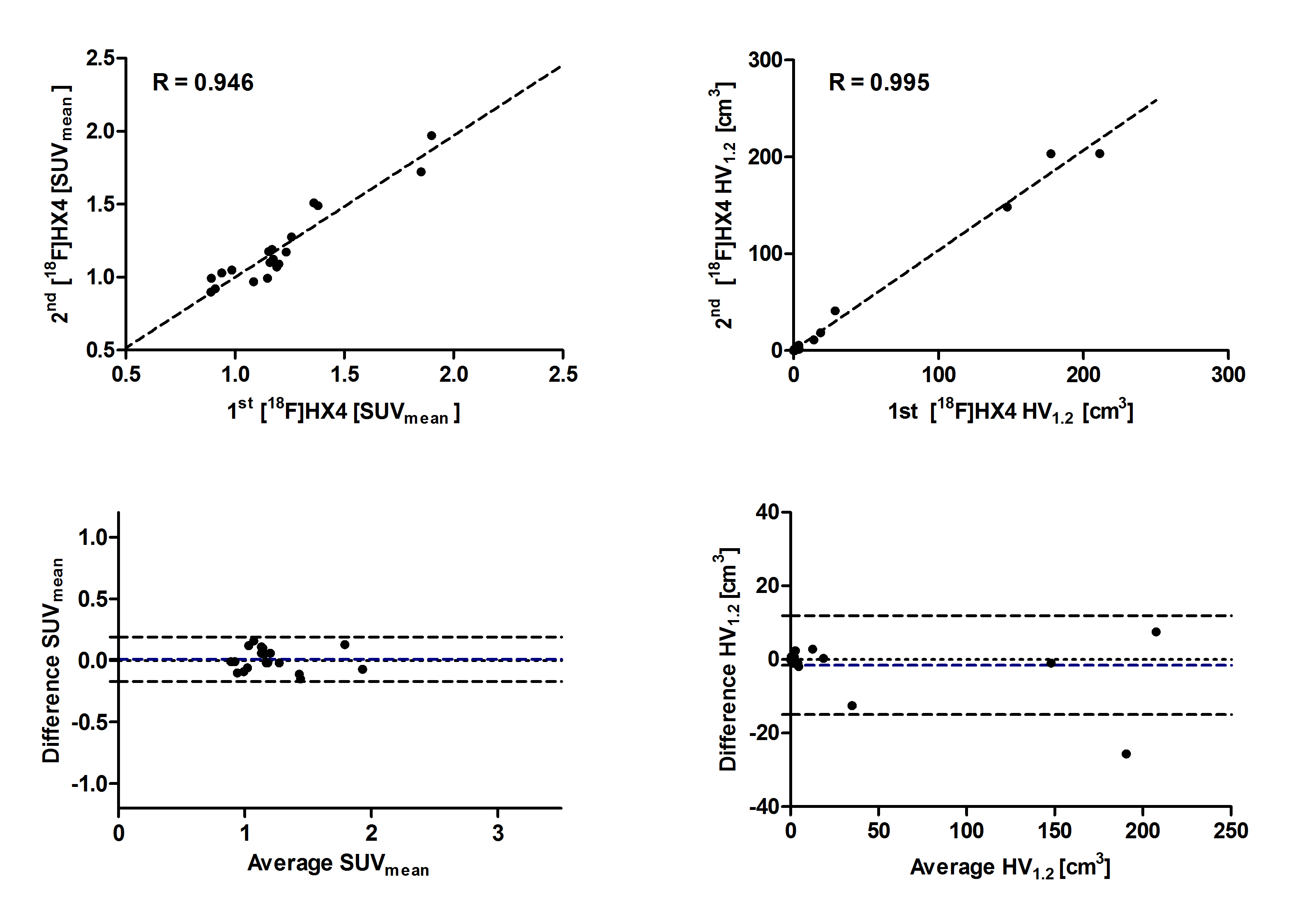

Supplement: Supplementary file 1 — Correlation and Bland-Altman plots of image parameters SUVmean and hypoxic tumor volume (HV1.2). (JPEG 740 kb) [file 259_2015_3100_Fig4_ESM.jpg]
