# Supplementary material for: Repeatability of hypoxia PET imaging using [18F]HX4 in lung and head and neck cancer patients: a prospective multicenter trial
Source: Eur J Nucl Med Mol Imaging. 2015 Jul 2;42(12):1840–9. doi: 10.1007/s00259-015-3100-z (PMC4589564; doi:10.1007/s00259-015-3100-z)
Supplement: Supplementary file 2 — (DOC 72 kb) [file 259_2015_3100_MOESM1_ESM.doc]

Supplementary Table: Repeatability of the [^18^F]HX4 hypoxic tumor volume and fraction using a threshold of 1.4 times background (HV_1.4_)

|  | HV_1.4_ [cm^3^] | | FHV_1.4_[%] | | |
| --- | --- | --- | --- | --- | --- |
| Patient ID | Scan 1 | Scan 2 | Scan 1 | Scan 2 | |
| Lung Cancer | | | | | |
| 01 | 3.05 | 2.33 | 3.43 | | 2.37 |
| 02 | 77.43 | 54.67 | 21.42 | | 14.21 |
| 03 | 0.00 | 0.00 | 0.00 | | 0.00 |
| 04 | 96.00 | 121.34 | 38.16 | | 44.73 |
| 05 | 1.60 | 3.52 | 1.83 | | 6.86 |
| 06 | 0.45 | 0.25 | 1.95 | | 1.32 |
| 07 | 0.05 | 0.15 | 0.49 | | 1.00 |
| 08 | 1.39 | 1.79 | 15.11 | | 18.75 |
| 09 | 0.00 | 0.00 | 0.00 | | 0.00 |
| Mean±SD (Lung) | 20.0±38.1 | 20.5±41.8 | 9.2±13.3 | | 9.9±14.7 |
| Head and Neck Cancer | | | | | |
| 10 | 0.00 | 0.00 | 0.00 | | 0.00 |
| 11 | 0.00 | 0.00 | 0.00 | | 0.00 |
| 12 | 0.00 | 0.00 | 0.00 | | 0.00 |
| 13 | 0.00 | 0.00 | 0.00 | | 0.00 |
| 14 | 0.06 | 0.00 | 0.36 | | 0.00 |
| 15 | 0.13 | 1.41 | 0.41 | | 5.20 |
| 16 | 0.31 | 0.52 | 4.42 | | 8.68 |
| 17 | 175.37 | 161.63 | 70.68 | | 63.50 |
| 18 | 0.00 | 0.00 | 0.00 | | 0.00 |
| 19 | 12.06 | 18.74 | 17.66 | | 26.15 |
| Mean±SD (HN) | 18.8±55.1 | 18.2±50.7 | 9.4±22.2 | | 10.2±17.4 |
| Mean±SD (total) | 19.4±46.6 | 19.3±45.4 | 9.3±18.0 | | 10.4±20.4 |
